# On Analytical Modeling of Hopping Transport of Charge Carriers and Excitations in Materials with Correlated Disorder

**DOI:** 10.1021/acs.jpclett.4c00097

**Published:** 2024-02-28

**Authors:** Anna Yu. Saunina, Libai Huang, Vladimir R. Nikitenko, Oleg V. Prezhdo

**Affiliations:** † Department of Condensed Matter Physics, National Research Nuclear University, Moscow Engineering Physics Institute (MEPhI), Kashirskoe Shosse 31, 115409 Moscow, Russia; ‡ Department of Chemistry, Purdue University, West Lafayette, Indiana 47907, United States; § Department of Chemistry, University of Southern California, Los Angeles, California 90089, United States

## Abstract

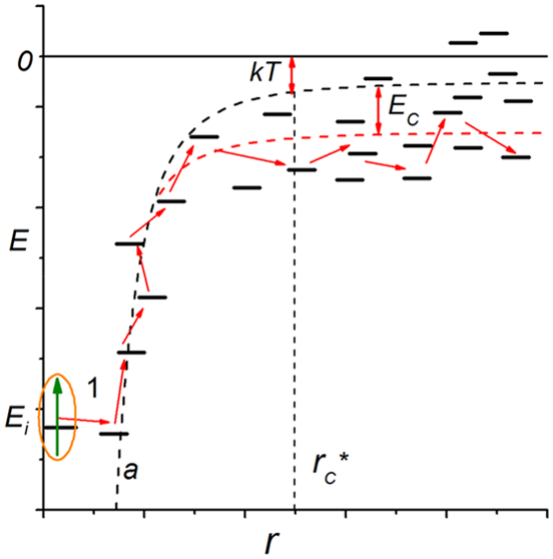

Spatial-energy correlations strongly influence charge and exciton transport in weakly ordered media such as organic semiconductors and nanoparticle assemblies. Focusing on cases with shorter-range interparticle interactions, we develop a unified analytic approach that allows us to calculate the temperature and field dependence of charge carrier mobility in organic quadrupole glasses and the temperature dependence of the diffusion coefficient of excitons in quantum dot solids. We obtain analytic expressions for the energy distribution of hopping centers, the characteristic escape time of charge/exciton from the energy well stemming from energy correlations around deep states, and the size of the well. The derived formulas are tested with Monte Carlo simulation results, showing good agreement and providing simple analytic expressions for analysis of charge and exciton mobility in a broad range of partially ordered media.

Organic semiconductors, quantum dot (QD) solids, and other partly ordered condensed matter systems are actively studied as promising materials for solar energy, light-emitting, and other optoelectronic devices.^1−5^ Transport of both charge and excitation has a large effect on the performance of such devices. Despite the differences in chemical composition, it has been shown that, from the point of view of transport, disordered organic semiconductors and QD solids have much in common: energetic disorder can lead to localization of electron and hole states and, consequently, to the hopping transport mechanism.^6,7^ It is known that spatial-energy correlations significantly affect transport in organic semiconductors, especially materials composed of molecules with large dipole moments, when the correlations are the most long-range (dipole glasses).^8,9^ Typically, hopping transport in materials with correlated disorder is modeled by numerical Monte Carlo (MC) methods,^8,9^ but recently an analytic approach has been developed.^10^ Correlations arise due to electrostatic interactions (in the case of dipole glass, interactions of charges with randomly oriented dipoles). The effect of correlations arising from weaker interactions, the range of which is shorter, is less clear. In this work, an analysis has been carried out for two such cases: charge transfer in quadrupole glasses^11,12^ and diffusion of triplet excitons in QD solids, using a unified formalism. The energy density of states (DOS), which controls the hopping transport of excitons in QD solids, is also calculated.

It is known that excitons in QD solids can diffuse over distances much greater than the size of a QD.^13−15^ Note that the transfer of triplet excitons between QDs occurs according to the Dexter mechanism, which is a charge exchange mechanism; therefore, the hopping transport model can also be considered for triplet excitons.^16^ Large intrinsic dipole moments of QDs^17−19^ create energy correlations. In previous work,^10^ a model was proposed for calculating the mobility of charge carriers in organic dipole glasses, which considered a multistep process of charge carrier exit from a potential well caused by energy correlations. Description of the hopping transport of triplet excitons in QD solids can be obtained by using a similar approach; however, it is necessary to estimate the corresponding spatial size of the potential well and determine its shape. One can consider an exciton as a pointlike dipole with the moment, *d* = *er*_ex_ and its interaction with randomly oriented dipole moments of QDs, *p*, situated at the nodes of a simple cubic lattice with constant *a*. The lattice constant corresponds to the distance between the centers of QDs, such that *a* = *d*_QD_ + *l*, where *d*_QD_ is the QD diameter and *l* is the edge-to-edge distance (ligand length). The density of energy states in this system can be calculated as^11^
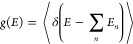
1where *E*_*n*_ is the interaction energy between the exciton (pointlike dipole) and the dipole at the node *n*:
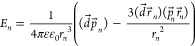
2The summation is carried out over all nodes except the one at which the exciton is located. Averaging over all random vector directions ***r*_*n*_** and ***p*_*n*_** gives
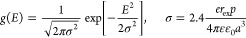
3Thus, the presence of large permanent dipole moments of QDs leads to the formation of Gaussian disorder for exciton states, and the scale of the disorder depends on the size of the exciton, the dipole moments of the QDs, and the dielectric constant of the medium.

Determination of the shape of the potential well, formed around a deep energy state, requires calculation of the correlation function in the system:^11^
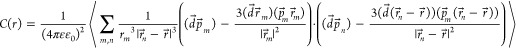
4Carrying out statistical averaging and replacing summation with integration gives
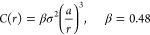
5This type of correlation function is also typical for quadrupole glasses,^11^ with a slight difference in the coefficient β (for quadrupole glasses, β = 0.5). The form of the correlation function and the energy distribution suggests that the conditional probability of a state situated at a distance *r* from the initial state with energy *E*_*i*_ to have the energy *E* has the following form:^9^
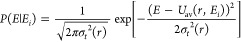
6
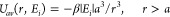
7
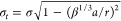
8This means that deep states with energies *E*_*i*_ ≈ −σ^2^/*kT* controlling the conductance in a weak external electric field are surrounded by a potential well, defined by eq 7. One can define the spatial size of this well by the condition *U*_av_(*r*_C_*, *E*_*i*_) = *kT*, in analogy to the Coulomb radius (the Onsager radius):
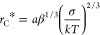
9The diffusion coefficient is calculated according to the ubiquitous (for the low-field limit) expression:
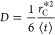
10where ⟨*t*⟩ is the average time required for a carrier to escape from the potential well. Thus, as in ref (10), the release of a carrier from a deep state is considered as a multistep process. One can distinguish two stages: (1) the first jump from the initial state to the nearest neighbor (most likely having the same energy due to the strong correlations), with a frequency ν; (2) drift-diffusion over a region of size *r*_C_^*^, which depends on the depth of the well (i.e., on the energy of the initial state *E*_i_). Note that the escape probability is low. The scheme of the process is shown in Figure 1.

**Figure 1:**
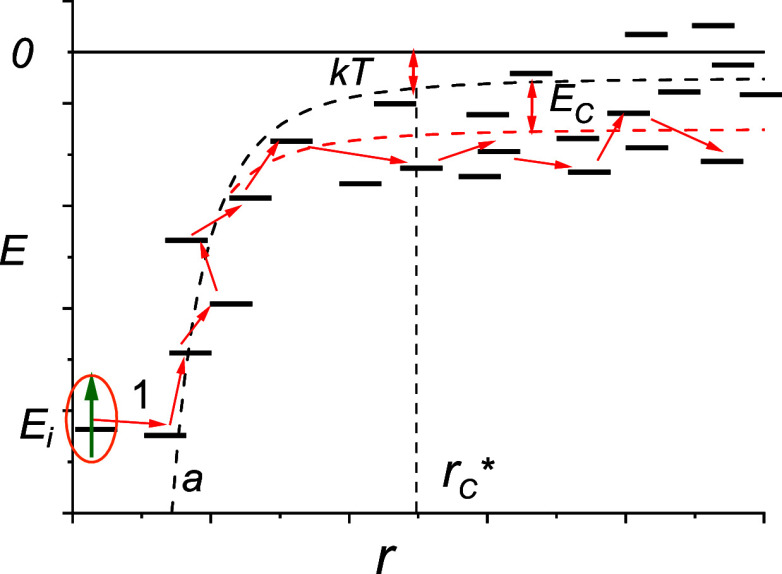
Scheme of exciton escape from a potential well formed around the deep state *E*_*i*_. The dashed line shows the energy dependence given by eq 7, and the number 1 corresponds to a typical first jump from the initial state.

The typical number of attempts to escape is inversely proportional to the probability of a release after the first jump. The average escape time can be calculated as^10^
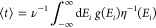
11where ν = ν_0_ exp (−2γ*r*_0_), and the typical tunnelling hopping length *r*_0_ is defined by the condition *U*(*r*_0_) ≈ *U*_av_(*r*_0_) = *E*_*i*_. For the triplet transport in QD solids, *r*_0_ = β^1/3^*l*. The probability of leaving the well (η) is calculated as the separation probability of a pair of particles interacting according to eq 7. To calculate the separation probability, it is necessary to solve the Smoluchowski equation with potential (eq 7) in the space of initial separations *r*_0_:^20,21^
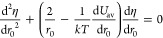
12with the boundary conditions:
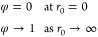
Using the method from previous work,^21^ we obtain
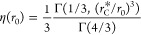
13Since the main contribution to the integral described by eq 11 is made by deep states, we will use the asymptote for the Γ function in expression described by eq 13. We also take into account, similarly to work,^10^ that the transport level *E*_C_ < 0 (calculated according to previous work^22^), which lies below the maximum of the Gaussian distribution, reduces the height of the energy barrier that the carrier must overcome, see Figure 1, as noted in ref (23). Thus, energetic disorder increases the separation probability:
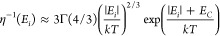
14Equations 11 and 14 give
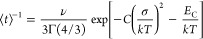
15
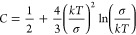
16Finally, from eqs 9, 10, 15, and 21, we obtain
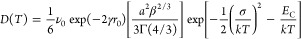
17

In eqs 15 and 17, *E*_C_ is the effective transport level,^22^*E*_C_ = *E*_tr_ – 2γ*l*, where the tunneling length (*l*) replaces the intersite distance (*a*), because hopping sites are not pointlike, in contrast to previous work;^22^*E*_tr_ is defined from the following equation:
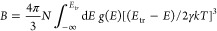
18where *B* = 2.77, *N* = *a*^–3^.

For CdSe QDs with diameter *d*_QD_ = 3 nm, the reported parameters are the following: dielectric constant, ε ≈ 6.2;^24^ and QD dipole moment, *p* ≈ 25 D.^18^ These parameters along with the short ligands (*l* = 0.5 nm) give the disorder scale σ ≈ 20 meV (see eq 3). The proposed approach assumes that the condition *r*_C_^*^ > *a* is met. Estimation of the size of the potential well from eq 9 gives *r*_C_^*^ > 2*a* at *T* < 120 K, indicating that the effect of correlations is strongly pronounced at low temperatures. The obtained result is qualitatively consistent with the conclusions of ref (16), which showed that, at low temperatures, the transport of triplet excitons in organic materials is more influenced by disorder and, at high temperatures, by polaronic effects.

As for the hopping parameters, the values of the inverse localization radius (γ) of ∼0.5 Å^–1^ were reported,^6^ depending on the chemical structure of the ligand molecule. The value of the phonon frequency ν_0_ = 7.5 × 10^11^ s^–1^ for the 3 nm CdSe QD was reported in the calculations.^25^ Using these parameters, we derived the absolute values for the diffusion coefficient *D* = 1.5 × 10^–4^ sm^2^/s for temperature *T* = 100 K and *D* = 6.7 × 10^–4^ sm^2^/s for *T* = 298 K, which are qualitatively consistent with the data of some experimental works. For example, previous work^13^ has reported a value of *D* ≈ 3 × 10^–4^ sm^2^/s at room temperature for core/shell CdSe/ZnCdS QDs. Note that the proposed model works best at low temperatures, at which experimental data are limited, making the comparison difficult.

Since the resulting form of the correlation function and the density of states are in good agreement with the formulas for charge transport in quadrupole organic glasses (see, for example, refs (11 and 12)), in order to verify the approach, the results of the calculations of the diffusion coefficient according to eq 11 were compared to the results of Monte Carlo simulation in the quadrupole glass model^12^ with the parameters typical for the disordered organic media: ε = 3, *p* = 3 D, σ = 0.078 eV, *a* = *a*_0_ = 1 nm (*a*_0_ is the intermolecular distance), γ*a* = 5, *r*_0_ = β^1/3^*a*_0_. This estimated value of σ is calculated for the quadrupole moment *Q* = *pr*_ex_, where quadrupoles are formed by two oppositely directed dipoles with moment *p*, located at a distance *r*_ex_ from each other, *r*_ex_*=* 1 nm. The estimation of the size of the potential well from eq 9 gives *r*_C_^*^ > 3*a*_0_ at *T* < 125 K. Figure 2 shows the good agreement with the Monte Carlo results, as well as with the empirical formula for the case of zero external electric field^12^ ln(*D*/*D*_0_) = −0.37(σ/*kT*)^2^ at temperatures below room temperature (*T* < 278 K) (even for *r*_C_^*^ ≈ *a*_0_). The difference with the Monte Carlo results for large values of σ/*kT* is explained by the fact that the data were taken for a weak but nonzero field, the influence of which on mobility increases along with σ/*kT*.

**Figure 2:**
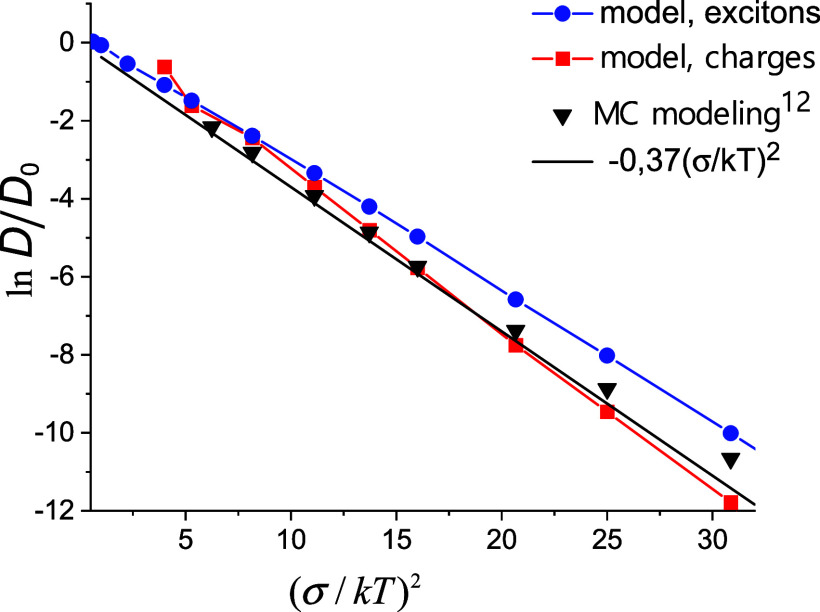
Temperature dependence of the diffusion coefficient for exciton transport in QD solids and charge transport in organic quadrupole glasses, in comparison with the MC simulation results;^12^*D*_0_ = .

In the first approximation, the temperature dependences of the diffusion coefficient both in the case of quadrupole glasses and QD solids can be expressed by the well-known for organic materials dependence,^8,12,26^ ln(*D*/*D*_0_) = −const(σ/*kT*)^2^. For the case of QD solids, one obtains const ≈ 0.34, whereas for the case of quadrupole glasses, const ≈ 0.42 is the same as in the Gaussian disorder model^26^ and is slightly different from ln(*D*/*D*_0_) = −0.37(σ/*kT*)^2^ (see Figure 2). The difference in the constants follows from the difference in the temperature dependences of *E*_C_, which, in turn, follows from the difference in the localization parameters used: γ*a*_0_ = 5 for the quadrupole glass and γ*l* = 2.25 for the QD solid.

We can also obtain the field dependence of the mobility by using the field-dependent effective temperature concept (ETC) modified in this work. The essence of the ETC is that the electric field *F* reduces the activation energy of jumps by *eFh* (in a disordered medium and, in the case of a uniform field, the length scale *h* is the localization radius γ^–1^). Consequently, the jump frequency and mobility increase. Qualitatively, an increase in the temperature has the same effect. In the limit *T* = 0, one has *T*_eff_(0, *F*) ≈ *eFh*/*k*.^27^ For the general case, one obtains interpolation expressions such as
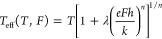
19where λ is a numerical constant, and usually, *n* = 2.^28,29^ When a particle leaves a potential well, the electric field is nonuniform and the process is multistep. By analogy with the Onsager model, in which the quantum yield depends on the parameter *eFr*_0_/2*kT*,^20^ one assumes that the exponent *n* in eq 19 depends on this parameter. According to the fitting data (see Figure 3), the exponent *n* increases from 1 to 4 with increasing field *F*. The distance *r*_0_ is the spatial scale. Thus, in eq 19,
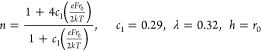
20

**Figure 3:**
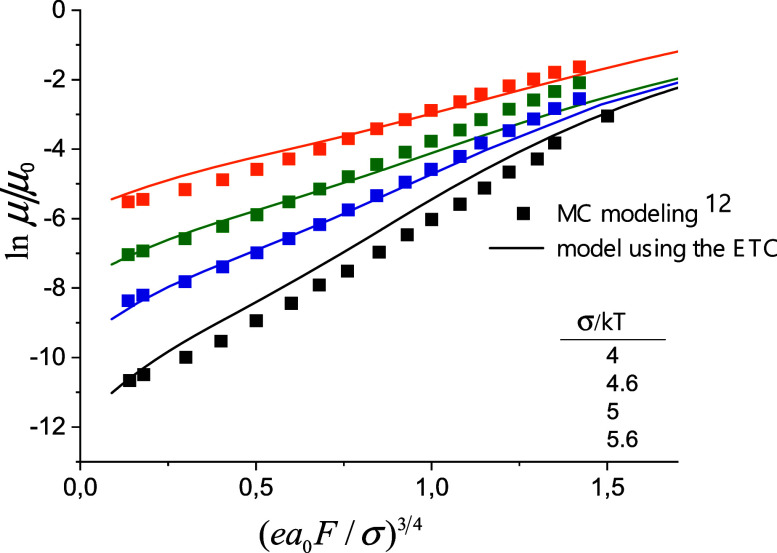
Field dependence of charge carrier mobility in quadrupole glasses, in comparison with the MC simulation data.^12^

The mobility for a weak electric field is calculated from eq 17, using the Einstein relation:
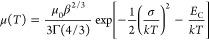
21with μ_0_ = . Here, *E*_C_ = *E*_tr_ – 2γ*a*, the same as in previous work.^22^ We obtain the field dependence of mobility in a quadrupole glass by replacing the temperature *T* in eq 21 with the effective temperature, *T*_eff_(*T*, *F*). The field dependence of mobility with the above-mentioned parameters for quadrupole glasses, obtained using the modified ETC, are compared in Figure 3 with the Monte Carlo simulation data for quadrupole glasses from ref (12). The agreement is good, especially at moderate field strengths. Equation 21 gives the correct value and field dependence of hole mobility in nonpolar organic material 4,4′-bis(*N*-carbazolyl)-1,1′-biphenyl (CBP), as measured in previous work,^30^ providing the independently determined^31^ values σ = 0.125 eV and μ_0_ = 0.8 cm^2^ V^–1^ s^–1^. We obtain the inverse localization radius γ = 2.5/*a*_0_ from fitting; hence, ν_0_ = 10^15^ s^–1^ from eq 21. This is an example of the application of our model for the estimation of physical parameters that are difficult to measure for a given material.

In summary, the energy distribution of hopping centers that control the triplet exciton diffusion has been calculated in this work. This distribution is in the form of a Gaussian function. The energy disorder is spatially correlated. It arises due to the electrostatic interaction of the exciton dipole moment with the randomly oriented molecular dipole moments. When the width of the DOS was calculated, other possible contributions to disorder have not been taken into account, primarily the spread in QD sizes and spatial disorder in QD positions. This approach is justified by the fact that one aims to achieve the most ordered QD-solid structures for applications. On the other hand, random orientation of QD dipoles is assumed, while their partial ordering can reduce disorder. Nevertheless, the resulting expression for the width of the Gaussian DOS appears to be a lower estimate.

The characteristic escape time of an exciton from the energy well, which arises as a result of energy correlations around deep states, has been calculated together with the characteristic size of the well. These quantities determine the magnitude and temperature dependence of the exciton diffusion coefficient. A simple analytical expression for the diffusion coefficient of triplet excitons in QD solids has been obtained. This expression (see eq 17) is reminiscent of the expression for the coefficient of hopping diffusion of charge carriers in disordered organic semiconductors. The proposed approach has been tested by comparison with the known^12^ results of MC modeling of charge carrier mobility in organic quadrupole glasses (where a similar type of correlation exists). Using the same approach, an analytic model of the diffusion coefficient and mobility for quadrupole glasses has been developed and justified by comparison with the experimental data.^30,31^
